# Prognostic Value of the Endothelial Activation and Stress Index (EASIX) in Critically Ill Patients With Ischemic Stroke: A MIMIC‐IV Database Analysis

**DOI:** 10.1002/brb3.71708

**Published:** 2026-08-20

**Authors:** Wen Bai, Manli Tao, Shaofei Li, Wei Shi, Kun Jiang, Ling Tian

**Affiliations:** ^1^ Department of Critical Care Medicine Yantai Yuhuangding Hospital Yantai Shandong China; ^2^ Department of Neurology Yantai Yuhuangding Hospital, Qingdao University Yantai Shandong China; ^3^ Department of Critical Care Medicine The Affiliated Brain Hospital of Nanjing Medical University Nanjing China

**Keywords:** EASIX, endothelial dysfunction, incremental predictive value, ischemic stroke, MIMIC‐IV, risk stratification

## Abstract

**Background:**

Systemic endothelial dysfunction is a critical driver of poor outcomes in ischemic stroke. We evaluated the prognostic value of the endothelial activation and stress index (EASIX) in critically ill stroke patients.

**Methods:**

We analyzed 3063 patients with ischemic stroke from the MIMIC‐IV database. The natural log‐transformed EASIX (lnEASIX) was calculated from admission LDH, creatinine, and platelets. The primary outcome was designated as 28‐day mortality, with secondary outcomes including in‐hospital, 90‐day, and 180‐day mortality. Multivariable logistic and Cox proportional hazards regression, restricted cubic splines (RCS), and advanced clinical utility analyses (NRI, IDI, and decision curve analysis) were utilized.

**Results:**

Evaluated as a continuous variable, a per‐unit increase in lnEASIX was independently associated with an elevated risk for the primary endpoint of 28‐day mortality (OR 1.47, 95% CI 1.36–1.59, *p < *0.001), as well as all secondary mortality endpoints. RCS analysis confirmed a strictly linear, monotonic dose–response relationship (*p* for nonlinearity > 0.05). While lnEASIX demonstrated moderate standalone discrimination (AUC 0.64–0.67, comparable to SOFA), integrating it with the baseline SOFA score yielded significant incremental predictive value (NRI 0.208, IDI 0.011; both *p < *0.01) and superior net clinical benefit. Subgroup analysis revealed consistent prognostic utility across most strata, though the impact was markedly more pronounced in patients without pre‐existing cancer (*p* for interaction < 0.05).

**Conclusion:**

LnEASIX is a simple, robust, and continuous predictor of short‐ and long‐term mortality in critically ill patients with ischemic stroke. Integrating this zero‐cost biomarker with established intensive care scoring systems provides significant incremental value for early prognostic risk stratification.

## Graphical abstract

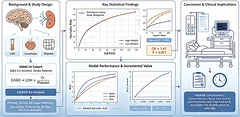

## 1 Introduction

Ischemic stroke represents a leading cause of global disability and mortality, with a significant proportion of patients requiring intensive care due to severe neurological deficits or systemic complications (Naghavi et al. 2025; Feigin et al. 2024). Despite advancements in reperfusion therapies, the prognosis for critically ill stroke patients remains heterogeneous and often poor (Naghavi et al. 2025; Feigin et al. 2024). Traditional prognostic indicators, such as the National Institutes of Health Stroke Scale (NIHSS), the Glasgow Coma Scale (GCS), and the sequential organ failure assessment (SOFA), are indispensable in clinical practice; however, these tools primarily focus on either focal neurological impairment or established end‐organ failure (Koton et al. 2026; Lusk et al. 2023; Sluis et al. 2025; Vincent et al. 1996). They may lack the sensitivity to capture the early, occult systemic biological stress and microvascular endotheliopathy that frequently drive secondary brain injury and multi‐organ dysfunction in the post‐stroke phase (Balistreri et al. 2024; Liu et al. 2024).

The neurovascular unit, particularly the cerebral endothelial interface, is the primary target of the ischemic cascade (Fangma et al. 2025; Goertz et al. 2023). Acute cerebral ischemia triggers not only localized blood–brain barrier disruption but also a profound systemic inflammatory response (Acioglu and Elkabes 2025; Boltze and Perez‐Pinzon 2022). This “brain–body” crosstalk leads to widespread endothelial activation, characterized by a pro‐thrombotic state, impaired vasoreactivity, and microcirculatory exhaustion (Bu et al. 2019; Fangma et al. 2025). Recent evidence suggests that this systemic endotheliopathy is not merely a bystander effect but a central mediator of post‐stroke complications, including infectious complications, myocardial injury, and acute kidney injury, all of which substantially worsen the clinical trajectory (Bochaton et al. 2022; Jafari et al. 2021).

In view of the critical role of systemic endothelial dysfunction, the endothelial activation and stress index (EASIX) has recently been proposed as a robust, objective surrogate for assessing the intensity of systemic endothelial stress (Luft et al. 2017). Derived from three readily available laboratory markers—lactate dehydrogenase (LDH), creatinine, and platelet count—EASIX integrates markers of cellular hypoxia, renal perfusion, and consumptive coagulopathy into a single, pragmatic score (Frenking et al. 2024; Liu et al. 2025). While its prognostic utility was initially validated in the setting of hematopoietic stem cell transplantation and subsequently in hematological malignancies, the biological rationale for EASIX—reflecting the nexus of inflammation, endothelial damage, and microvascular thrombosis—aligns closely with the pathophysiology of acute ischemic stroke (Luft et al. 2017; Merz et al. 2019).

Despite the theoretical overlap between endothelial stress and stroke progression, the specific prognostic relevance of EASIX in the context of critical care stroke management has not been systematically explored (Balistreri et al. 2024; Nitzsche et al. 2021; Real et al. 2024). It remains undetermined whether this simplified composite index can offer incremental predictive value beyond existing neurological and systemic severity scales.

Therefore, the present study aimed to investigate the relationship between the natural log‐transformed EASIX (lnEASIX)—evaluated primarily as a continuous variable—and adverse outcomes in a large‐scale, real‐world cohort of critically ill patients with ischemic stroke, with 28‐day all‐cause mortality explicitly designated as the primary clinical endpoint. We hypothesized that an elevated lnEASIX would serve as a robust, independent predictor of both short‐ and long‐term mortality, and that its integration into conventional prognostic models (such as the SOFA score) would significantly refine early risk stratification, providing a more holistic and actionable assessment of the systemic stress burden in this vulnerable population.

## 2 Methods

### 2.1 Study Design and Data Source

This investigation was designed as a retrospective cohort study utilizing the MIMIC‐IV (v3.1) database, a high‐granularity clinical repository from the Beth Israel Deaconess Medical Center. We extracted de‐identified longitudinal data for critically ill patients hospitalized between 2008 and 2019. Access was authorized under record (15577060; Wen Bai) after institutional ethics approval and completion of the required human subjects protection training. This study was conducted and reported in accordance with the STROBE (strengthening the reporting of observational studies in epidemiology) statement.

### 2.2 Subject Selection

Participants were identified based on a primary discharge diagnosis of acute ischemic stroke, defined by the International Classification of Diseases, Ninth Revision, Clinical Modification (ICD‐9‐CM) codes 433.01, 433.11, 433.21, 433.31, 433.81, 433.91, 434.01, 434.11, and 434.91, or the Tenth Revision (ICD‐10) codes I63.0 through I63.9 (including subclassifications such as I63.81 and I63.89)

Eligible patients were those aged 18 years or older at their initial ICU admission.

**FIGURE 1:**
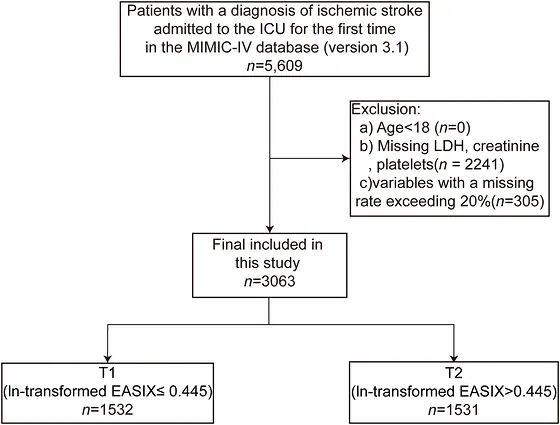
Study flowchart and patient selection. Flow diagram illustrating the detailed screening and selection process of critically ill patients with ischemic stroke included in the final analysis. EASIX, endothelial activation and stress index.

Exclusionary criteria involved: (1) inadequate laboratory data for calculating the EASIX index (specifically LDH, creatinine, and platelets missing within 24 h of admission); and (2) candidate variables with an overall missing rate exceeding 20% were entirely excluded from the analysis. For the remaining baseline covariates with a missing rate of less than 20%. A detailed illustration of the study cohort selection process is presented in Figure 1.

#### 2.2.1 Variable Definitions and EASIX Calculation

The EASIX was defined as [LDH (U/L) ∖times Creatinine (mg/dL)] / Platelets (10^9^/L). To normalize the typically right‐skewed distribution of raw values, we utilized the natural log‐transformed version (lnEASIX) for all parametric testing.

Baseline covariates were harvested within the first 24‐h window of ICU stay, encompassing:

*Physiological markers*: Heart rate, MBP, respiratory rate, SpO_2_, and temperature.

*Severity scores*: SOFA, LODS, and the Charlson comorbidity index (CCI).

*Comorbid conditions*: Such as atrial fibrillation, congestive heart failure, and renal impairment.

### 2.3 Clinical Outcomes

The primary endpoint of this study was explicitly designated as 28‐day all‐cause mortality, reflecting the critical acute and subacute prognostic window for severe ischemic stroke.

Secondary endpoints included in‐hospital mortality, 90‐day mortality, and 180‐day mortality to evaluate short‐ and long‐term outcomes. Secondary endpoints focused on temporal survival post‐admission, specifically at 28, 90, and 180 days, providing a comprehensive view of both acute and subacute prognosis.

### 2.4 Statistical Framework

Statistical analyses were executed using STATA (v18.0) and R (v4.4.1).

Missing data were handled using a two‐step strategy. Patients with missing values in the primary components required to calculate the EASIX score (LDH, creatinine, or platelets) were excluded. For other baseline covariates with a missing rate of less than 20%, data were assumed to be missing at random (MAR). To minimize bias and maintain statistical power, we performed multiple imputation by chained equations (MICE) using the “mice” package (version 3.16.0) in R. Five imputed datasets were generated, and the results were pooled according to Rubin's rules.

#### 2.4.1 Baseline Comparisons

In primary analyses, lnEASIX was evaluated strictly as a continuous variable. To verify trend consistency and clinical utility, patients were further categorized into tertiles based on their lnEASIX values. Intergroup differences were evaluated using the Pearson's χ^2^ test (or Fisher's exact test, where appropriate) for categorical data and the Kruskal–Wallis test for non‐normal continuous variables.

#### 2.4.2 Regression Modeling

The independent prognostic significance of lnEASIX was examined through three‐stage hierarchical multivariable logistic regression models (reporting Odds Ratios, ORs) and parallel Cox proportional hazards regression models (reporting Hazard Ratios, HRs) to account for time‐to‐event data. The models were constructed with progressive adjustment for potential confounders as follows:

*Model 1*: Unadjusted (crude model), evaluating the direct association between lnEASIX and mortality outcomes without controlling for any covariates.

*Model 2*: Adjusted for baseline demographic characteristics and initial vital signs to account for the patients' fundamental physiological states upon ICU admission. Specifically, covariates included age at admission, gender, race, mean heart rate, mean blood pressure (MBP), mean respiratory rate, mean temperature, and mean SpO2.

*Model 3 (Fully Adjusted Model)*: Adjusted for all covariates in Model 2, plus a comprehensive set of pre‐existing comorbidities. Specifically, the additionally incorporated variables included diabetes, chronic liver disease, cancer, hypertension, atrial fibrillation, myocardial infarction, congestive heart failure, peripheral vascular disease, dementia, and chronic pulmonary disease. (Note: Paraplegia and baseline renal disease were explicitly excluded from the models to prevent collider stratification bias and collinearity with the EASIX components, respectively).

To verify the absence of significant collinearity, variance inflation factors (VIF) were calculated for all covariates in Model 3, with VIF values < 1.5 confirming no substantial multicollinearity. Given the simultaneous evaluation of four mortality endpoints, a Bonferroni correction was applied to adjust for multiple comparisons. Consequently, statistical significance for the multivariable regression analyses was strictly defined as a two‐sided *p*‐value < 0.0125 (calculated as 0.05/4).

#### 2.4.3 Non‐Linear and Survival Analysis

Restricted cubic splines (RCS), fully adjusted for the covariates defined in Model 3, were employed to characterize the dose–response relationship between lnEASIX and mortality. Time‐to‐event data stratified by lnEASIX tertiles were visualized via Kaplan–Meier curves and assessed using the log‐rank test.

#### 2.4.4 Sensitivity Analysis via IPTW

To robustly mitigate baseline confounding and further validate the independent prognostic value of lnEASIX, an inverse probability of treatment weighting (IPTW) approach was employed as a sensitivity analysis. First, propensity scores were estimated using a multivariable logistic regression model that incorporated all baseline covariates utilized in our fully adjusted Model 3 (excluding variables directly related to the EASIX calculation). Subsequently, stabilized weights were applied to balance the distribution of these covariates between the high and low lnEASIX groups (stratified by the median for this specific analysis). The adequacy of covariate balance post‐weighting was evaluated using absolute standardized mean differences (SMDs), with an SMD < 0.1 indicating an excellent balance. Finally, IPTW‐adjusted Kaplan–Meier survival curves were generated, and temporal survival differences were compared using a weighted log‐rank test.

#### 2.4.5 Model Validation

We evaluated the index's discriminative power via receiver operating characteristic (ROC) analysis. Specific area under the curve (AUC) values, sensitivities, specificities, and optimal clinical cutoff values were determined by maximizing the Youden index. Furthermore, the C‐statistics (AUC) of the multivariable logistic regression models were calculated, and DeLong's test was utilized to compare the discriminative performance sequentially across the three models. The predictive performance of the nomogram was evaluated using both discrimination and calibration. Calibration was assessed using calibration curves, which plot the nomogram‐predicted probabilities against the actual observed survival probabilities evaluated by the Kaplan–Meier method. To reduce over‐optimism, internal validation was performed using 1000 bootstrap resamples.

#### 2.4.6 Clinical Utility and Incremental Value

To robustly verify the incremental predictive value and clinical utility of lnEASIX beyond established critical care systems, we performed net reclassification improvement (NRI), integrated discrimination improvement (IDI), and decision curve analysis (DCA). These analyses evaluated the net benefit and reclassification accuracy when lnEASIX was integrated with baseline SOFA, LODS, and GCS scores.

#### 2.4.7 Robustness Check

Stratified analyses (forest plots) were conducted to ensure the consistency of findings across various clinical subgroups.

To provide a quantitative and intuitive tool for personalized clinical application, a predictive nomogram was constructed based on the optimized multivariable model. Variables incorporated into the nomogram were selected based on clinical relevance and their independent prognostic value, which included lnEASIX, age, SOFA score, and CCL. The individualized risk probabilities for 28‐day, 90‐day, and 180‐day mortality can be estimated by summing the points assigned to each specific predictor. The predictive accuracy and absolute risk estimation of this nomogram were subsequently evaluated using calibration curves with 1000 bootstrap resamples for internal validation.

## 3 Results

### 3.1 Baseline Characteristics

This study included 3063 patients with first‐admission ischemic stroke. For descriptive baseline comparisons, patients were categorized into low (*n* = 1532) and high (*n* = 1531) lnEASIX groups based on the median value. Patients in the High lnEASIX group were more likely to be male and Black (*p < *0.001), presenting with significantly greater disease severity upon ICU admission, as indicated by elevated SOFA, LODS, and Charlson comorbidity scores (all *p < *0.001). Furthermore, this group exhibited a higher burden of pre‐existing conditions, most notably chronic renal disease, heart failure, diabetes, and myocardial infarction (all *p < *0.001) (Table 1).

**TABLE 1:** Baseline characteristics of patients stratified by lnEASIX.

Characteristic	Overall	Low	High	*p* value
	*n = *3063	*n = *1532	*n = *1531	
Gender [Female (n, %)]	1472(48.06)	819(53.46)	653(42.65)	*< *0.001
Admission_ageAge (year, IQR)	70.85 (60.46–80.35)	70.05(59.25–80.20)	71.29(61.38–80.42)	0.081
Race (n, %)				*< *0.001
White	1734(56.61)	926(60.44)	808(52.78)	
Black	454(14.82)	187(12.21)	267(17.44)	
Other	875(28.57)	419(27.35)	456(29.78)	

Vital signs				
Heart rate (beats/min, IQR)	82.48(72.35–94.09)	81.15(71.28–91.61)	83.80(73.79–96.72)	*< *0.001
Resp rate (breath/min, IQR)	19.19(17.09–21.86)	18.80(16.86–21.31)	19.59(17.44–22.69)	*< *0.001
Mean blood pressure (mmHg, IQR)	82.68(74.43–92.38)	85.78(77.50–95.26)	79.66(72.04–88.48)	*< *0.001
Temperature (°C, IQR)	36.87(36.67–37.15)	36.87(36.69–37.10)	36.87(36.64–37.20)	0.773
SpO2(%, IQR)	97.18(95.84–98.52)	97.03(95.77–98.36)	97.35(95.91–98.67)	*< *0.001
Scores				
Charlson comorbidity index (IQR)	7(5–9)	6(4–8)	8(6–10)	*< *0.001
SOFA (IQR)	4(2–7)	3(1–5)	6(4–9)	*< *0.001
LODS (IQR)	4(2–7)	3(2–5)	6(4–8)	*< *0.001
GCS (IQR)	15(14–15)	15(14–15)	15(14–15)	*< *0.001
Comorbidities				
Atrial fibrillation (n, %)	1262(41.20)	567(37.01)	695(45.40)	*< *0.001
Paraplegia (n, %)	1070(34.93)	636(41.51)	434(28.35)	*< *0.001
Chronic hepatic disease (n, %)	261(8.52)	69(4.50)	192(12.54)	*< *0.001
Peripheral vascular disease (n, %)	440(14.37)	186(12.14)	254(16.59)	*< *0.001
Myocardial infarct (n, %)	681(22.23)	233(15.21)	448(29.26)	*< *0.001
Hypertension (n, %)	1355(44.24)	851(55.55)	504(32.92)	*< *0.001
Diabetes (n, %)	1176(38.39)	496(32.38)	680(44.42)	*< *0.001
Heart failure (n, %)	1055(34.44)	405(26.44)	650(42.46)	*< *0.001
Dementia (n, %)	257(8.39)	117(7.64)	140(9.14)	0.132
Chronic pulmonary disease (n, %)	646(21.09)	316(20.63)	330(21.55)	0.529
Chronic renal disease (n, %)	821(26.80)	177(11.55)	644(42.06)	*< *0.001
Cancer (n, %)	368(12.01)	162(10.57)	206(13.46)	0.014
Laboratory tests				
WBC (10^9^/L, IQR)	9.90(7.30–13.20)	9.60(7.30–12.60)	10.20(7.40–14.00)	*< *0.001
RBC (10^9^/L, IQR)	3.44(2.92–4.08)	3.75(3.18–4.33)	3.17(2.76–3.72)	*< *0.001
Hemoglobin (g/dL, IQR)	10.00(8.50–12.00)	10.80(9.00–12.70)	9.40(8.20–11.00)	*< *0.001
Platelet (10^9^/L, IQR)	213.00(153.00–292.00)	250.00(197.00–321.00)	177.00(117.00–245.00)	*< *0.001
ALT (U/L, IQR)	22.00(14.00–42.00)	19.00(13.00–31.00)	27.00(15.00–57.75)	*< *0.001
AST (U/L, IQR)	29.00(19.00–49.00)	24.00(18.00–35.00)	38.00(24.00–72.00)	*< *0.001
Creatinine (mg/dL, IQR)	1.05(0.80–1.60)	0.85(0.70–1.05)	1.50(1.05–2.55)	*< *0.001
INR (s, IQR)	1.20(1.10–1.40)	1.20(1.10–1.40)	1.30(1.10–1.50)	*< *0.001
PT (sec, IQR)	13.40(12.10–15.70)	13.00(11.85–14.80)	13.90(12.50–16.78)	*< *0.001
APTT (sec, IQR)	31.50(27.60–44.00)	30.70(27.20–40.80)	32.30(27.90–47.80)	*< *0.001
Chloride (mEq/L, IQR)	103.00(100.00–107.00)	103.00(100.00–107.00)	104.00(99.00–108.00)	0.613
Potassium (mEq/L, IQR)	4.00(3.70–4.40)	4.00(3.70–4.30)	4.10(3.80–4.50)	*< *0.001
Calcium (mEq/L, IQR)	8.70(8.20–8.70)	8.80(8.30–9.20)	8.60(8.10–9.10)	*< *0.001
Sodium (mEq/L, IQR)	140.00(137.00–143.00)	140.00(137.00–143.00)	140.00(137.00–142.00)	0.870
ICU stay (days, IQR)	3.65(1.74–7.88)	2.99(1.56–6.62)	4.29(1.98–9.22)	*< *0.001
Hospital stay (days, IQR)	11.80(6.35–21.64)	9.89(5.64–18.11)	13.74(7.41–24.44)	*< *0.001
Outcomes				
Primary outcome				
28‐day mortality (n, %)	895 (29.22)	308 (20.10)	587 (38.34)	*< *0.001
Secondary outcomes				
In‐hospital mortality (n, %)	617(20.14)	195(12.73)	422(27.56)	*< *0.001
90‐day mortality (n, %)	1073 (35.03)	391 (25.52)	682 (44.55)	*< *0.001
180‐day mortality (n, %)	1180 (38.52)	441 (28.79)	739 (48.27)	*< *0.001

Abbreviations: ALT, alanine aminotransferase; APTT, activated partial thromboplastin time; AST, aspartate aminotransferase; BUN, blood urea nitrogen; GCS, Glasgow Coma scale; INR, international normalized ratio; IQR, interquartile range; LODS, logistic organ dysfunction system; PT, prothrombin time; RBC, red blood cell; SOFA, sequential organ failure assessment; SpO_2_, peripheral capillary oxygen saturation; WBC, white blood cell.

Laboratory analyses revealed more severe systemic derangements in the high lnEASIX group, characterized by lower hemoglobin and platelet counts, alongside elevated LDH, creatinine, liver enzymes, and coagulation parameters (all *p < *0.001). Consequently, the high lnEASIX group experienced prolonged ICU and hospital stays. Crucially, they demonstrated significantly higher mortality rates for the primary endpoint (28‐day mortality), as well as all secondary endpoints (in‐hospital, 90‐day, and 180‐day mortality; all *p < *0.001).

### 3.2 Association Between lnEASIX and Mortality Outcomes

To evaluate the independent prognostic value of lnEASIX for adverse outcomes, we primarily modeled lnEASIX as a continuous variable using three‐stage hierarchical multivariable logistic regression (Table 2). Potential multicollinearity among the covariates included in the final multivariable models was rigorously assessed; all VIF values (ranging from 1.04 to 1.41) (Table S1) were well below the predefined threshold of 5, indicating the absence of significant multicollinearity.

**TABLE 2:** Multivariable logistic regression analysis of lnEASIX for mortality outcomes. Rates of 28‐day, in‐hospital, and 90‐day all‐cause mortality stratified by lnEASIX groups.

Outcomes	Events / Total (n/N)	Model 1		Model 2		Model 3	
		OR (95% CI)	*p* value	OR (95% CI)	*p* value	OR (95% CI)	*p* value
28‐day mortality	895/3063	1.60 (1.49‐1.71)	< 0.001	1.52 (1.40–1.64)	< 0.001	1.47 (1.36–1.59)	< 0.001
In‐hospital mortality	617/3063	1.62 (1.51‐1.75)	< 0.001	1.47 (1.35–1.59)	< 0.001	1.44 (1.32–1.56)	< 0.001
90‐day mortality	1073/3063	1.52 (1.42‐1.62)	< 0.001	1.43 (1.33–1.54)	< 0.001	1.37 (1.26–1.48)	< 0.001
180‐day mortality	1180/3063	1.51 (1.42‐1.61)	< 0.001	1.43(1.32‐1.54)	*< *0.001	1.47 (1.36‐1.59)	*< *0.001

*Note: p*‐values were interpreted using a Bonferroni‐corrected threshold of *p < *0.0125 due to the testing of four mortality endpoints.

In the unadjusted model (Model 1), a per‐unit increase in lnEASIX was significantly associated with an increased risk of the primary endpoint, 28‐day mortality (OR 1.60, 95% CI 1.49–1.71, *p* < 0.001), as well as in‐hospital, 90‐day, and 180‐day mortality (all *p < *0.001). After adjusting for demographic characteristics and vital signs (Model 2), the associations remained robust. Crucially, in the fully adjusted model (Model 3)—which further incorporated pre‐existing baseline comorbidities—lnEASIX remained a strong, independent predictor for 28‐day mortality (OR 1.47, 95% CI 1.36–1.59, *p < *0.001). This independent predictive value was consistently maintained across all secondary endpoints, including in‐hospital (OR 1.44, *p < *0.001), 90‐day (OR 1.37, *p < *0.001), and 180‐day mortality (OR 1.47, *p < *0.001), underscoring its enduring prognostic utility.

Furthermore, parallel Cox proportional hazards regression models were conducted to account for time‐to‐event data. This dual‐method approach confirmed our findings, demonstrating that an elevated lnEASIX (evaluated both as a continuous variable and across tertiles) remained a robust and independent predictor of 28‐day, 90‐day, and 180‐day mortality even under the strict Bonferroni‐corrected threshold (all *p < *0.0125; Table S2).

**FIGURE 2:**
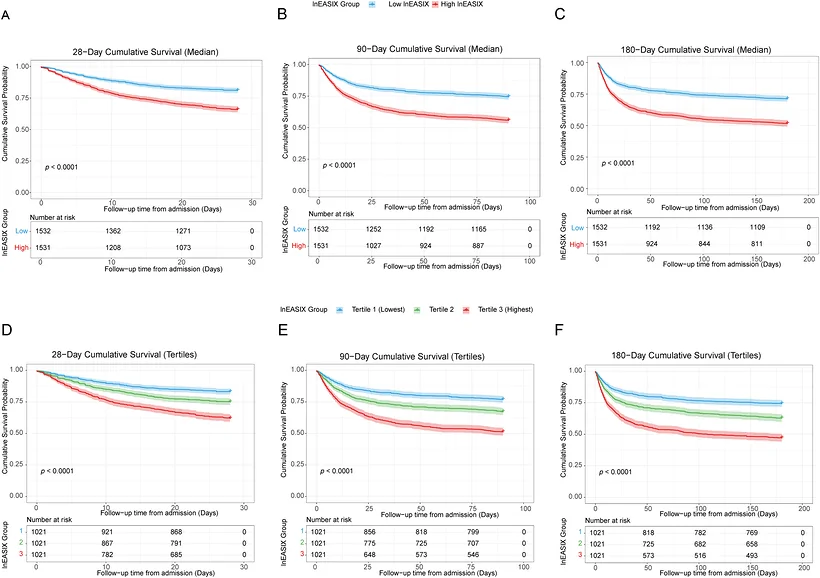
Kaplan–Meier cumulative survival analyses according to lnEASIX median and tertiles. (A–C) Kaplan–Meier curves for (A) 28‐day, (B) 90‐day, and (C) 180‐day cumulative all‐cause mortality from admission, stratified by the median lnEASIX value (low vs. high). (D–F) Kaplan–Meier curves for (D) 28‐day, (E) 90‐day, and (F) 180‐day cumulative all‐cause mortality from admission, stratified by lnEASIX tertiles (Tertile 1 [lowest] to Tertile 3 [highest]). All survival times were calculated from the day of ICU admission. Differences in survival probabilities among the stratified groups were assessed using the log‐rank test.

To visualize the impact of lnEASIX on temporal prognosis, Kaplan–Meier cumulative survival curves were constructed from the day of admission. Initially, patients were dichotomized based on the median lnEASIX value. As shown in Figure 2A–C, patients in the high lnEASIX group (red line) exhibited significantly lower cumulative survival probabilities across 28‐day, 90‐day, and 180‐day observation periods compared to those in the low lnEASIX group (blue line). To further validate and visualize the dose–response relationship, patients were subsequently stratified into tertiles (Tertile 1 [lowest] to Tertile 3 [highest]). This analysis revealed a strict, dose‐dependent divergence in survival (Figure 2D–F): patients in the highest lnEASIX tertile (Tertile 3, red line) experienced the worst prognosis, followed sequentially by the intermediate (Tertile 2, green line), and lowest tertiles (Tertile 1, blue line). The log‐rank test confirmed statistically significant differences among the groups in both stratification methods (all *p < *0.001). These visualization results perfectly align with the multivariable regression findings, reinforcing elevated lnEASIX as a robust, continuous indicator of poor short‐ and long‐term survival in critically ill patients with ischemic stroke.

To further rigorously validate the independent prognostic value of lnEASIX and minimize the impact of baseline confounding, an IPTW analysis was performed as a supplementary robustness check. Following IPTW adjustment, all baseline covariates included in the propensity score model (such as demographics, vital signs, and pre‐existing comorbidities) achieved excellent balance between the high and low lnEASIX groups, with all absolute SMDs falling well below the 0.1 threshold (Figure S1; Table S3). As clinically expected, variables directly related to the calculation of the EASIX score—namely platelets and creatinine—as well as comprehensive severity scores encompassing these organ functions (e.g., SOFA and LODS), remained appropriately unadjusted. Under this rigorously balanced cohort, the IPTW‐adjusted Kaplan–Meier survival curves consistently demonstrated that patients with high lnEASIX experienced significantly lower overall survival probabilities across 28‐day, 90‐day, and 180‐day follow‐up intervals (all weighted *p < *0.001; Figure S2). This robust sensitivity analysis strongly reinforces our multivariable regression findings, confirming that the prognostic stratification value of the EASIX index is independent of baseline confounding factors.

### 3.3 Dose–Response Relationship Between lnEASIX and Outcome Events

**FIGURE 3:**
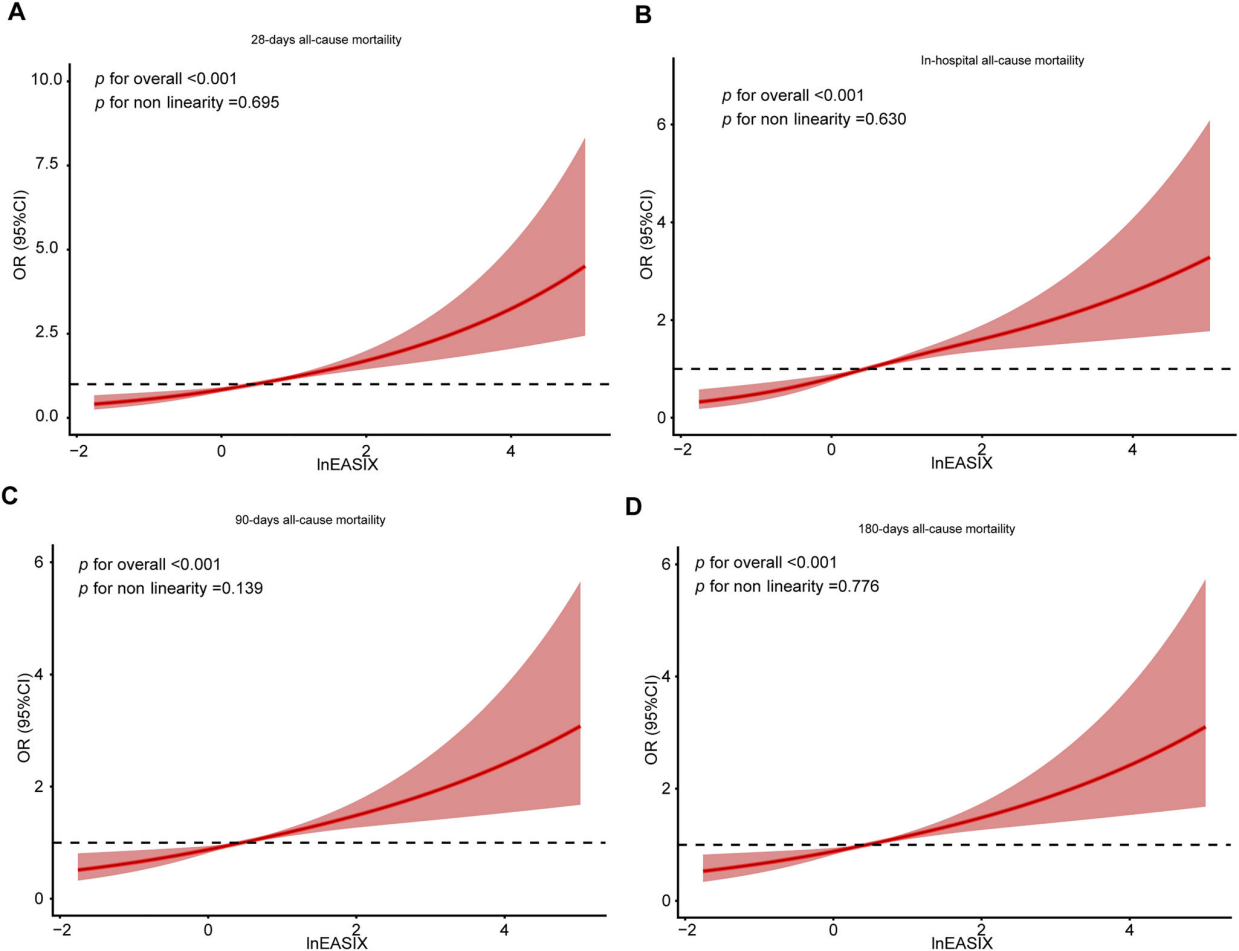
Restricted cubic spline (RCS) analyses of lnEASIX and mortality outcomes in critically ill patients with ischemic stroke. (A) Association between lnEASIX and in‐hospital mortality. (B) Association between lnEASIX and 28‐day all‐cause mortality after hospital discharge. (C) Association between lnEASIX and 90‐day all‐cause mortality after hospital discharge. (D) Association between lnEASIX and 180‐day all‐cause mortality after hospital discharge. Models were adjusted for covariates included in the fully adjusted model.

To further explore the dose–response relationship between lnEASIX and mortality outcomes, RCS analyses were performed (Figure 3). After adjusting for the confounding variables included in Model 3, a significant linear association was observed between lnEASIX and the risk of in‐hospital, 28‐day, 90‐day, and 180‐day all‐cause mortality (all *p* for overall < 0.001). As illustrated in Figures 3A–D, the risk of mortality across all timeframes increased steadily with rising lnEASIX values. The tests for nonlinearity yielded non‐significant results (*p* for nonlinearity = 0.630, 0.695, 0.139, and 0.776, respectively), confirming that the association between lnEASIX and mortality is predominantly monotonic and linear, rather than characterized by a specific non‐linear inflection point.

Because a traditional non‐linear statistical threshold does not exist for this dataset, we addressed the clinical need for risk stratification by calculating optimal clinical cut‐off values using the Youden index from ROC curves. The optimal lnEASIX cut‐offs for predicting in‐hospital, 28‐day, 90‐day, and 180‐day mortality were identified as 1.01, 0.67, 0.67, and 0.63, respectively (Table S4). Patients presenting with lnEASIX scores exceeding these pragmatic clinical thresholds may require more aggressive monitoring, reinforcing the role of lnEASIX as a robust and continuous predictor of prognosis in critically ill patients with ischemic stroke.

### 3.4 Model Discrimination and Calibration

To evaluate the discriminative performance of the hierarchical models, C‐statistics (equivalent to the area under the ROC curve) were calculated and compared using DeLong's test (Table 3). Model 1, which included lnEASIX alone, demonstrated moderate discriminative ability across all temporal endpoints (e.g., C‐statistic 0.66, 95% CI: 0.64–0.68 for 28‐day mortality). The sequential addition of demographic characteristics and vital signs in Model 2 significantly improved the model's discriminative power (C‐statistic 0.73, *p < *0.001 vs. Model 1). Furthermore, the fully adjusted Model 3, which incorporated extensive baseline comorbidities, yielded the highest C‐statistics across all endpoints (ranging from 0.74 to 0.75), showing statistically significant improvements over Model 2 (all *p < *0.05). This confirms that a comprehensive model incorporating lnEASIX and baseline clinical variables provides robust prognostic accuracy.

**TABLE 3:** Discriminative performance and incremental predictive value of hierarchical multivariable models for mortality outcomes.

Outcomes	Model 1	Model 2		Model 3	
	C‐statistic (95% CI)	C‐statistic (95% CI)	*p* value	C‐statistic (95% CI)	*p* value
28‐day mortality	0.66 (0.64–0.68)	0.73 (0.71–0.75)	< 0.001	0.74 (0.72–0.76)	< 0.001
In‐hospital mortality	0.67 (0.65–0.70)	0.73 (0.71–0.75)	< 0.001	0.74 (0.72–0.76)	0.0401
90‐day mortality	0.64 (0.62–0.67)	0.73 (0.71–0.74)	< 0.001	0.75 (0.73–0.77)	< 0.001
180‐day mortality	0.64 (0.62–0.66)	0.73 (0.71–0.75)	< 0.001	0.75 (0.74–0.77)	< 0.001

*Note*: The discriminative ability of each model is expressed as the C‐statistic (equivalent to the Area under the receiver operating characteristic curve). Model 1 is an unadjusted model incorporating lnEASIX alone. Model 2 is adjusted for lnEASIX along with demographic characteristics (age, gender, and race) and admission vital signs. Model 3 (the fully adjusted model) includes all variables from Model 2 plus pre‐existing baseline comorbidities (diabetes, chronic liver disease, cancer, hypertension, atrial fibrillation, myocardial infarct, congestive heart failure, peripheral vascular disease, dementia, and chronic pulmonary disease). The *p*‐values denote statistical significance of the incremental improvement in model discrimination, calculated using DeLong's test. Specifically, the *p*‐value in the Model 2 column evaluates the comparison between Model 2 and Model 1; the *p*‐value in the Model 3 column evaluates the comparison between Model 3 and Model 2.

Abbreviation: CI, confidence interval.

**FIGURE 4:**
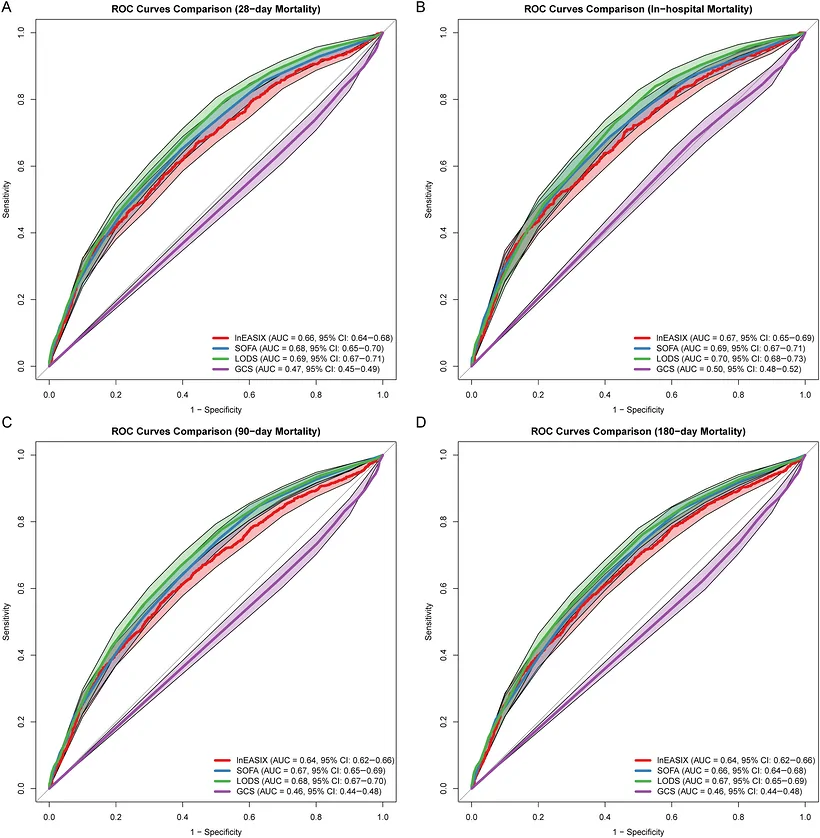
Receiver operating characteristic (ROC) curves of lnEASIX for mortality prediction**. (**A) ROC curve of lnEASIX for predicting in‐hospital all‐cause mortality. (B) ROC curve of lnEASIX for predicting 28‐day all‐cause mortality after hospital discharge. (C) ROC curve of lnEASIX for predicting 90‐day all‐cause mortality after hospital discharge. (D) ROC curve of lnEASIX for predicting 180‐day all‐cause mortality after hospital discharge.

Furthermore, isolated ROC analysis was conducted to compare the baseline discriminative ability of lnEASIX against established intensive care scoring systems (Figure 4). The results demonstrated that lnEASIX possessed moderate standalone discriminative ability for predicting the primary endpoint of 28‐day mortality (AUC 0.66). Consistent predictive capabilities were also observed across all secondary endpoints, including in‐hospital (AUC 0.67), 90‐day (AUC 0.64), and 180‐day mortality (AUC 0.64). Notably, the predictive performance of this single blood‐based biomarker was comparable to comprehensive clinical scores such as SOFA (AUC 0.66–0.69) and LODS (AUC 0.67–0.70), and was markedly superior to the GCS score (AUC 0.46–0.50). To facilitate individualized risk assessment in clinical practice, a predictive nomogram was developed incorporating lnEASIX alongside key established prognostic factors, specifically age, SOFA score, and CCL (Figure S3). By drawing a vertical line from each variable's value up to the “Points” axis, clinicians can calculate a total score, which corresponds to the estimated individual probability of 28‐day, 90‐day, and 180‐day mortality on the bottom axes.

**FIGURE 5:**
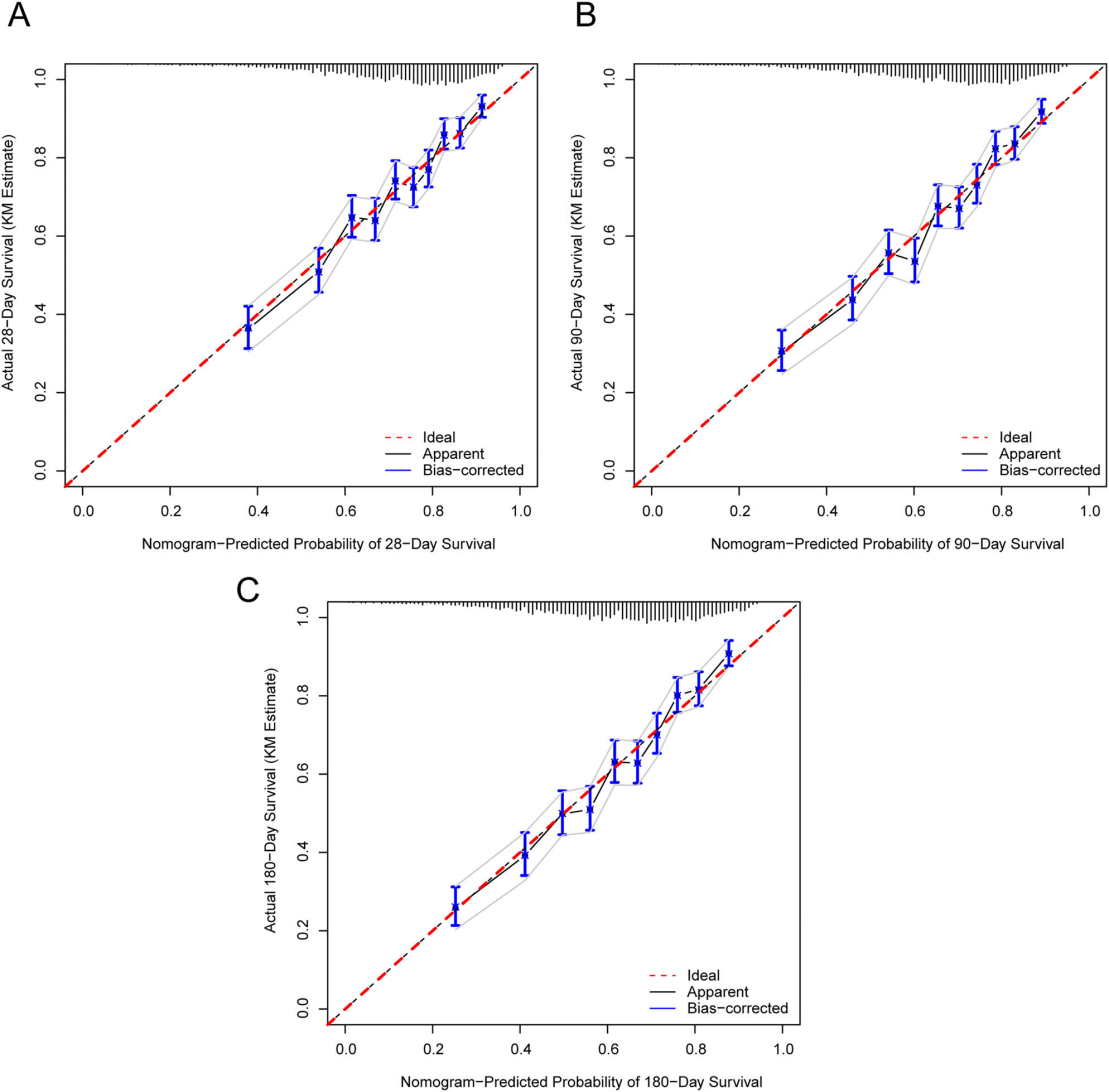
Calibration curves for internal validation of the prognostic model predicting 28‐day, 90‐day, and 180‐day survival. (A–C) Calibration plots comparing the nomogram‐predicted probabilities of survival with the actual observed survival (Kaplan–Meier estimates) at (A) 28 days, (B) 90 days, and (C) 180 days after admission. The x‐axis represents the predicted survival probability, while the y‐axis represents the actual survival rate. The red dashed line denotes a theoretically perfect prediction (ideal line, where predicted probability exactly matches actual outcome). The solid black line represents the apparent predictive accuracy of the model. The blue solid line with error bars illustrates the bias‐corrected calibration performance, rigorously calculated using 1000 bootstrap resamples for internal validation. The close alignment of the bias‐corrected lines with the ideal 45‐degree reference lines across all three timeframes indicates an optimal agreement and highly accurate absolute risk estimation, without significant overestimation or underestimation.

To verify predictive accuracy, calibration curves for the primary endpoint (28‐day survival), as well as secondary endpoints (90‐day and 180‐day survival), were generated using 1000 bootstrap resamples for internal validation (Figure 5). The bias‐corrected lines remained closely aligned with the 45‐degree ideal reference lines, indicating optimal agreement between the predicted probabilities and actual Kaplan–Meier observed survival. This demonstrates that the model provides highly accurate absolute risk estimates across all evaluated timeframes without significant overestimation or underestimation.

### 3.5 Incremental Predictive Value and Clinical Utility

Because a standalone biomarker with moderate discriminative ability is often insufficient to drive isolated clinical decisions, we rigorously evaluated the incremental predictive value of integrating lnEASIX with established ICU scoring systems.

**FIGURE 6:**
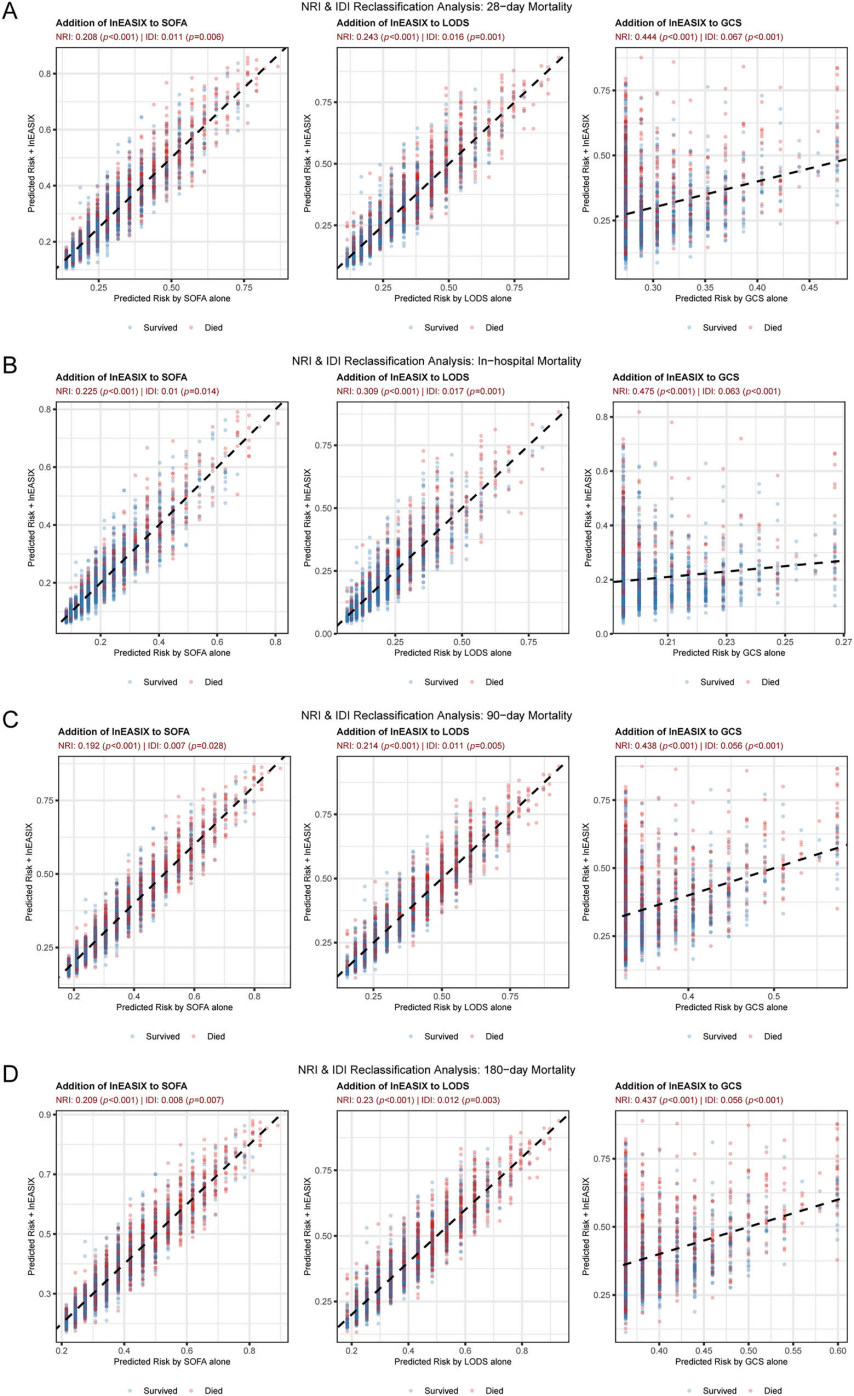
Reclassification improvement analyses (NRI and IDI) for the integration of lnEASIX with established intensive care scoring systems. (A–D) Scatter plots illustrating the changes in predicted mortality risk when lnEASIX is added to baseline clinical models (SOFA, LODS, and GCS) for predicting (A) 28‐day, (B) in‐hospital, (C) 90‐day, and (D) 180‐day mortality. The x‐axis represents the predicted risk calculated by the baseline clinical score alone, while the y‐axis represents the updated predicted risk after incorporating lnEASIX. Each dot represents an individual patient: red dots indicate patients who died (events), and blue dots indicate patients who survived (non‐events). The dashed black diagonal reference line represents no change in predicted risk. The net reclassification improvement (NRI) and integrated discrimination improvement (IDI) metrics, along with their respective *p*‐values, are displayed above each subplot. The consistently positive NRI and IDI values (all *p < *0.05) demonstrate that the addition of lnEASIX significantly reclassifies patients into more accurate risk categories, thereby substantially enhancing the overall prognostic performance of standard ICU scoring systems.

As illustrated by the reclassification analyses (Figure 6), the addition of lnEASIX to baseline SOFA, LODS, and GCS models resulted in highly significant improvements in risk stratification. For the primary 28‐day mortality endpoint, integrating lnEASIX with the SOFA score yielded a NRI of 0.208 (*p < *0.001) and an IDI of 0.011 (*p* = 0.006). Similar robust and statistically significant reclassification improvements were consistently observed across all other temporal endpoints when lnEASIX was added to the established clinical scores.

Finally, DCA was performed to assess the real‐world clinical applicability of this integration (Figure S4). The decision curves confirmed that combining lnEASIX with established scoring systems (e.g., SOFA + lnEASIX or LODS + lnEASIX) provided a consistently higher net clinical benefit across a wide range of threshold probabilities compared to relying on the baseline clinical scores alone. These findings confirm that lnEASIX, derived from routine and low‐cost laboratory tests, serves as a highly valuable and easily accessible adjunct for optimizing prognostic stratification in critically ill patients with ischemic stroke.

### 3.6 Subgroup Analysis

**FIGURE 7:**
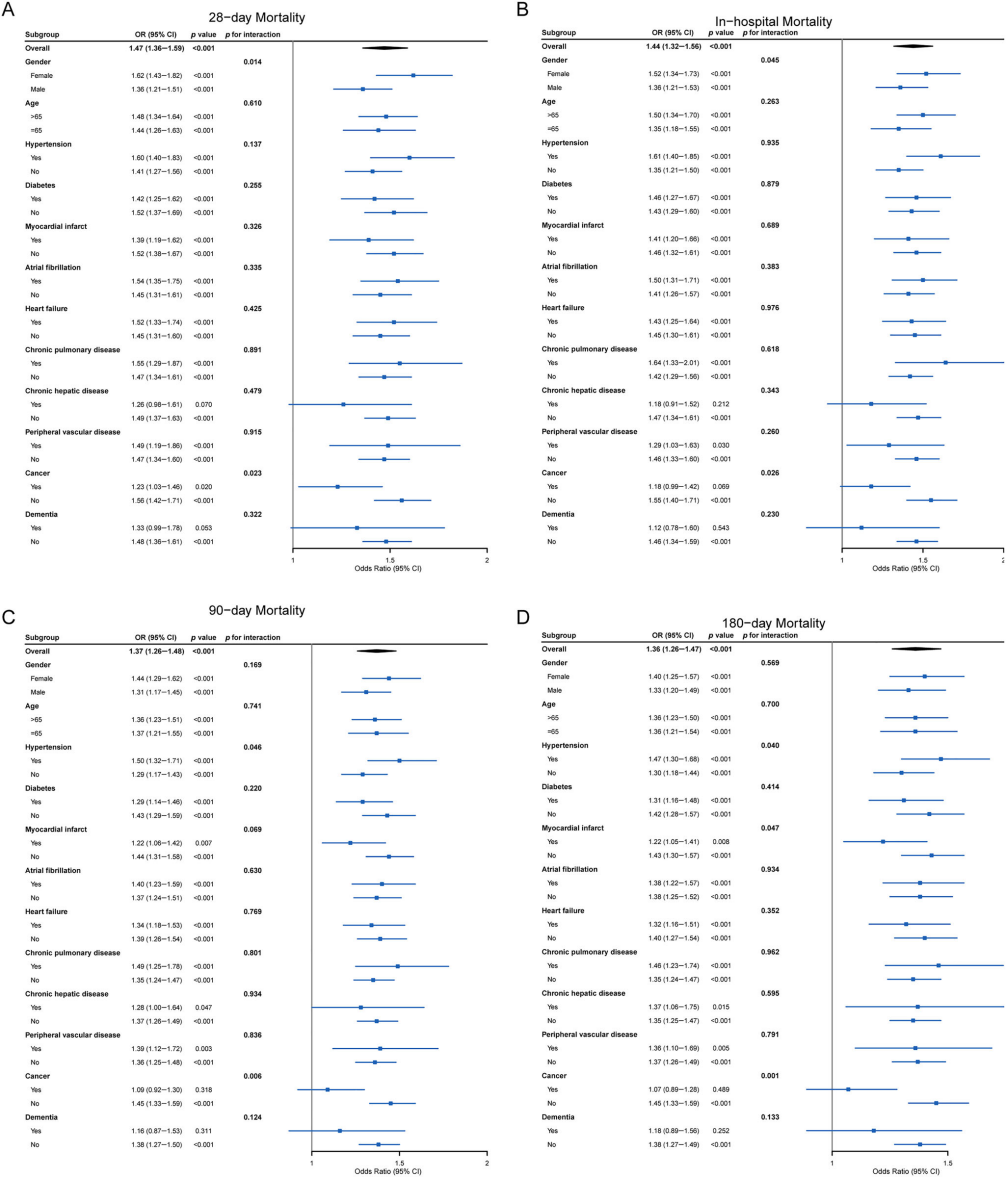
Subgroup analyses of the association between continuous lnEASIX and mortality outcomes. Forest plots illustrating the subgroup analyses for (A) 28‐day all‐cause mortality, (B) in‐hospital mortality, (C) 90‐day all‐cause mortality, and (D) 180‐day all‐cause mortality in critically ill patients with ischemic stroke. Hazard ratios (HRs) and 95% confidence intervals (CIs) for a per‐unit increase in lnEASIX were estimated using Cox proportional hazards regression models across various clinical strata. Interaction tests (*p* for interaction) were performed to assess effect modification by baseline characteristics. The consistently positive HRs (> 1) across the vast majority of subgroups demonstrate the robust and independent prognostic value of lnEASIX.

To assess the robustness of the prognostic value of lnEASIX as a continuous variable, comprehensive subgroup analyses were performed across multiple clinical strata (Figure 7). The association between a per‐unit increase in lnEASIX and elevated mortality risk remained consistently significant across the vast majority of subgroups for the primary endpoint of 28‐day mortality, as well as the secondary endpoints (in‐hospital, 90‐day, and 180‐day outcomes).

Notably, no significant interactions were observed for key demographic and clinical variables, such as age, diabetes, atrial fibrillation, heart failure, and peripheral vascular disease (all *p* for interaction > 0.05). This indicates that the predictive power of lnEASIX is highly stable and independent of these baseline characteristics.

However, significant interactions were consistently identified concerning pre‐existing cancer (*p* for interaction < 0.05 across all four temporal endpoints). In this subgroup, the association between lnEASIX and adverse outcomes was markedly more pronounced in patients without cancer (e.g., for 28‐day mortality, HR 1.56 in non‐cancer patients vs. HR 1.23 in cancer patients). In addition, minor interactions were observed for gender in short‐term outcomes, and hypertension and myocardial infarction in long‐term outcomes (180 days).

These findings suggest that while lnEASIX is a universally robust predictor, its relative risk‐stratification utility is particularly high in patients without a profound baseline burden of competing terminal conditions, such as cancer. Overall, the subgroup analysis reinforces the high reliability and broad clinical applicability of lnEASIX for prognostic assessment in critically ill patients with ischemic stroke.

## 4 Discussion

In this large‐scale retrospective cohort study, we demonstrated for the first time that the EASIX is a potent, independent, and continuous predictor of both short‐ and long‐term mortality in critically ill patients with ischemic stroke. Evaluated as a continuous variable, a per‐unit increase in lnEASIX was robustly associated with an elevated risk of the primary endpoint (28‐day mortality), as well as all secondary temporal endpoints (in‐hospital, 90‐day, and 180‐day mortality). Crucially, this monotonic dose–response relationship remained highly significant even after rigorously adjusting for demographics, admission vital signs, and extensive baseline comorbidities. Notably, the prognostic value of lnEASIX was consistent across the vast majority of clinical subgroups, although its relative predictive utility appeared particularly prominent in patients without a profound baseline burden of terminal conditions, such as cancer. Furthermore, we validated its real‐world clinical utility, demonstrating that integrating lnEASIX with established intensive care scoring systems (such as SOFA and LODS) yields significant incremental predictive value and net clinical benefit.

The prognostic utility of EASIX—a composite of LDH, creatinine, and platelets—lies in its ability to integrate the multifaceted pathophysiological cascade following acute ischemic stroke. LDH acts as a surrogate for systemic hypoxic burden and secondary tissue injury driven by the “brain–body” inflammatory response (X. Liu et al. 2026; Peh et al. 2026; Sun et al. 2026). Simultaneously, the inclusion of creatinine underscores the critical kidney–brain axis, where subtle microcirculatory dysfunction often precedes overt clinical deterioration (Lai et al. 2025). Furthermore, the platelet count reflects the “thrombo‐inflammatory” state and endothelial activation triggered by acute ischemia (Li et al. 2025). This unique pathophysiological focus explains the substantial incremental predictive value of lnEASIX when integrated with established clinical scoring systems. While traditional macroscopic scales such as the SOFA or GCS scores primarily quantify downstream, established organ dysfunction or localized neurological deficits, EASIX functions as a sensitive molecular “thermometer” that captures the upstream intensity of systemic endothelia (Merz et al. 2019). Because it reflects a completely distinct pathophysiological vector, EASIX perfectly complements these standard clinical assessments. By synergistically integrating metabolic disruption, hemodynamic instability, and microvascular thrombosis, EASIX provides a more holistic assessment of the systemic stress burden (Luft et al. 2017; Merz et al. 2019). Importantly, because the index is derived from routine, zero‐cost laboratory tests universally obtained upon ICU admission, it can be seamlessly automated into electronic health records as a continuous monitoring adjunct alongside SOFA, optimizing risk stratification without increasing the clinical workload.

One of the compelling findings of this study is the consistent prognostic value of lnEASIX, demonstrating moderate standalone discriminative ability with AUCs ranging from 0.64 to 0.67 across various temporal endpoints. While this level of standalone accuracy is comparable to—rather than independently superior to—established comprehensive intensive care scoring systems such as SOFA and LODS, its true clinical strength lies in its significant incremental predictive value when utilized as a supplementary adjunct. Furthermore, the RCS analysis revealed a strictly linear, monotonic dose–response relationship. This indicates that a traditional non‐linear “safe threshold” for endothelial stress does not exist; rather, any progressive increment in lnEASIX corresponds directly to a continuous and proportional increase in mortality risk.

Furthermore, our subgroup analysis revealed a significant interaction regarding pre‐existing cancer. The prognostic impact of lnEASIX was markedly more pronounced in patients without this condition (Al Heijani et al. 2025; Ganta and Annex 2021; Pasut et al. 2025; Sonobe and Kakinuma 2024). This suggests that in patients whose baseline systemic status is not heavily compromised by a severe competing terminal illness, the acute elevation of EASIX represents a sharper, unobstructed “biological signal” reflecting the severity of the primary stroke insult and subsequent endothelial stress. Conversely, in patients with a profound pre‐existing malignancy, this acute microvascular signal may be partially masked or diluted by the severe chronic systemic inflammation, baseline organ dysfunction, and high competing mortality risks inherently driven by the underlying cancer (Guo et al. 2025).

Traditional stroke prognosis often relies on neurological scales (e.g., GCS, NIHSS) (Poon et al. 2014; Sarraj et al. 2026). However, critically ill stroke patients frequently succumb to non‐neurological complications such as sepsis, myocardial injury, and renal failure (Ghannam et al. 2024; Khanzadeh et al. 2022; Schwarz et al. 2026). Our results suggest that lnEASIX provides a holistic view of systemic biological stress (Luft et al. 2017). By integrating parameters reflecting cellular injury, perfusion, and coagulation, EASIX offers a “liquid biopsy” of the endothelium's health, allowing clinicians to identify “high‐risk” patients who might appear stable on traditional neurological exams but are harboring severe systemic derangements.

Several limitations warrant consideration. First, as a single‐center retrospective cohort study utilizing the MIMIC‐IV database, potential selection bias cannot be entirely excluded. Although our comprehensive reclassification analyses (e.g., NRI, IDI) and DCA demonstrated significant incremental clinical utility, integrating lnEASIX with established ICU scoring systems requires rigorous prospective validation before routine clinical implementation. Second, as a composite index heavily reliant on LDH, the initial static EASIX score may be partially confounded by concurrent acute cardiac events. As observed in our baseline characteristics, myocardial infarction was more prevalent in the high lnEASIX group; because acute myocardial injury can cause a drastic systemic elevation of LDH entirely independent of stroke‐induced endothelial stress, the index should be interpreted cautiously in patients presenting with severe concurrent acute coronary syndromes. Furthermore, other unmeasured biological factors affecting LDH (e.g., in vivo hemolysis) were not evaluated. Third, our analysis was restricted to static data available within the first 24 h of ICU admission, meaning the dynamic changes of lnEASIX during the prolonged ICU stay were not captured. Future studies should explore whether the dynamic trajectory (i.e., “delta‐EASIX” or rate of change) provides even greater prognostic sensitivity.

In conclusion, lnEASIX is a simple, objective, and readily available biomarker that independently predicts short‐ and long‐term mortality in critically ill patients with ischemic stroke. While its standalone discriminative ability is moderate, its strictly linear dose–response association with adverse outcomes and its significant incremental predictive value when integrated with established tools (such as the SOFA score) make it a highly valuable adjunct for early risk stratification. Incorporating EASIX into clinical practice as a zero‐cost, continuous monitoring parameter could facilitate the early identification of patients at high risk for systemic microvascular complications, potentially guiding more personalized and intensive management strategies for this vulnerable population.

## Author Contributions

W. B. and M. T. conceived the study, designed the methodology, performed the data preprocessing and analysis, and drafted the manuscript. S. L. and W. S. contributed to the literature review, statistical validation. K. J. and L. T. contributed to the literature review, statistical validation, interpretation of results, and critical revision of the manuscript. All authors reviewed and approved the final version.

## Funding

The authors have nothing to report.

## Ethics Statement

This study was conducted in strict adherence to the ethical principles outlined in the Declaration of Helsinki. The utilization of the MIMIC‐IV database was formally approved by the institutional review boards (IRBs) of both the Massachusetts Institute of Technology and Beth Israel Deaconess Medical Center. Given that all data were de‐identified and publicly accessible via the MIMIC‐IV database, this retrospective observational study was exempt from requiring additional ethical approval and individual informed consent.

## Conflicts of Interest

The authors declare no conflicts of interest.

## Supporting information




**Supporting File 1**: brb371708‐sup‐0001‐SuppMat.docx

## Data Availability

The data supporting the findings of this study can be obtained from the corresponding author.
