# Supplementary material for: Prognostic Value of the Endothelial Activation and Stress Index (EASIX) in Critically Ill Patients With Ischemic Stroke: A MIMIC‐IV Database Analysis
Source: Brain Behav. 2026 Aug 20;16(8):e71708. doi: 10.1002/brb3.71708 (PMC13493690; doi:10.1002/brb3.71708)
Supplement: Supplementary file 1 [file BRB3-16-e71708-s001.docx]

**Supplementary Table S1**. Collinearity Diagnostics Using Generalized Variance Inflation Factors (GVIF) for Covariates in the Fully Adjusted Model (Model 3)

| Variables | GVIF | Df | GVIF^(1/(2*Df)) |
| --- | --- | --- | --- |
| lnEASIX | 1.2 | 1 | 1.1 |
| Age | 1.38 | 1 | 1.17 |
| Gender | 1.09 | 1 | 1.04 |
| Race | 1.12 | 2 | 1.03 |
| Mean heart rate | 1.26 | 1 | 1.12 |
| Mean MBP | 1.08 | 1 | 1.04 |
| Mean respiratory rate | 1.24 | 1 | 1.11 |
| Mean temperature | 1.1 | 1 | 1.05 |
| Mean SpO2 | 1.11 | 1 | 1.05 |
| Diabetes | 1.09 | 1 | 1.04 |
| Chronic liver disease | 1.11 | 1 | 1.05 |
| Cancer | 1.05 | 1 | 1.02 |
| Hypertension | 1.27 | 1 | 1.13 |
| Atrial fibrillation | 1.24 | 1 | 1.11 |
| Myocardial infarct | 1.11 | 1 | 1.05 |
| Congestive heart failure | 1.41 | 1 | 1.19 |
| Peripheral vascular disease | 1.04 | 1 | 1.02 |
| Dementia | 1.07 | 1 | 1.03 |
| Chronic pulmonary disease | 1.04 | 1 | 1.02 |

Note: GVIF, Generalized Variance Inflation Factor; Df, Degrees of freedom. The GVIF^(1/(2×Df)) metric is utilized to standardize the collinearity assessment for multi-categorical variables with more than one degree of freedom (such as Race). Values below the conventional threshold of 5 indicate the absence of significant multicollinearity. In this analysis, all calculated values range from 1.04 to 1.41, explicitly confirming that there is no significant collinearity among the demographic characteristics, vital signs, and comorbidities included in the final multivariable models

**Supplementary Table S2.** Multivariable Cox Proportional Hazards Regression Analysis of lnEASIX (Continuous and Tertiles) for 28-Day, 90-Day, and 180-Day Mortality

| **Outcomes** | **Predictor** | **Hazard Ratio (95% CI)** | **P-value** |
| --- | --- | --- | --- |
| 28-Day Mortality | | | |
| Continuous lnEASIX | Per 1-unit increase | 1.32 (1.21-1.44) | <0.001 |
| lnEASIX Tertiles | Tertile 1 (Lowest) | 1.00 (Reference) | — |
|  | Tertile 2 | 1.40 (1.03-1.90) | 0.031 |
|  | Tertile 3 (Highest) | 2.07 (1.54-2.77) | <0.001 |
| 90-Day Mortality | | | |
| Continuous lnEASIX | Per 1-unit increase | 1.23 (1.14-1.33) | <0.001 |
| lnEASIX Tertiles | Tertile 1 (Lowest) | 1.00 (Reference) | — |
|  | Tertile 2 | 1.30 (1.02-1.64) | 0.031 |
|  | Tertile 3 (Highest) | 1.92 (1.53-2.41) | <0.001 |
| 180-Day Mortality | | | |
| Continuous lnEASIX | Per 1-unit increase | 1.23 (1.15-1.32) | <0.001 |
| lnEASIX Tertiles | Tertile 1 (Lowest) | 1.00 (Reference) | — |
|  | Tertile 2 | 1.41 (1.14-1.74) | 0.002 |
|  | Tertile 3 (Highest) | 1.97 (1.60-2.42) | <0.001 |

**Note:** CI, Confidence Interval; HR, Hazard Ratio. Hazard Ratios were calculated using fully adjusted multivariable Cox proportional hazards regression models, adjusting for demographic characteristics, admission vital signs, and extensive baseline comorbidities (consistent with the covariates included in Model 3). To robustly control for the family-wise error rate across multiple temporal mortality endpoints, a strict Bonferroni-corrected threshold of P < 0.0125 was applied to determine statistical significance

**Supplementary Table S3.** Baseline characteristics of the study cohort stratified by median lnEASIX before and after Inverse Probability of Treatment Weighting (IPTW) adjustment.

| Characteristic | Before IPTW | | | | | After IPTW | | | | |
| --- | --- | --- | --- | --- | --- | --- | --- | --- | --- | --- |
|  | Low | High | p | test | SMD | Low | High | p | test | SMD |
|  | N=1510 | N=1464 |  |  |  | N=1522.72 | N=1457.17 |  |  |  |
| age (mean (SD)) | 68.85 (15.24) | 69.91 (14.63) | 0.053 |  | 0.071 | 69.49 (15.07) | 69.74 (14.80) | 0.697 |  | 0.017 |
| gender (Female) (%) | 808 (53.5) | 628 (42.9) | <0.001 |  | 0.214 | 726.2 (47.7) | 706.4 (48.5) | 0.709 |  | 0.016 |
| race group (%) |  |  | <0.001 |  | 0.176 |  |  | 0.992 |  | 0.005 |
| BLACK | 184 (12.2) | 258 (17.6) |  |  |  | 227.4 (14.9) | 216.5 (14.9) |  |  |  |
| WHITE | 909 (60.2) | 774 (52.9) |  |  |  | 847.0 (55.6) | 814.5 (55.9) |  |  |  |
| other | 417 (27.6) | 432 (29.5) |  |  |  | 448.4 (29.4) | 426.2 (29.2) |  |  |  |
| heart rate (mean (SD)) | 82.18 (15.09) | 85.99 (16.72) | <0.001 |  | 0.239 | 84.12 (15.89) | 83.93 (16.27) | 0.783 |  | 0.012 |
| resp rate (mean (SD)) | 19.32 (3.50) | 20.39 (4.19) | <0.001 |  | 0.277 | 19.87 (3.75) | 19.91 (3.97) | 0.811 |  | 0.01 |
| mbp (mean (SD)) | 86.95 (12.85) | 81.44 (12.31) | <0.001 |  | 0.437 | 84.41 (12.43) | 84.33 (13.20) | 0.891 |  | 0.006 |
| temperature (mean (SD)) | 36.92 (0.43) | 36.91 (0.60) | 0.871 |  | 0.006 | 36.92 (0.43) | 36.92 (0.59) | 0.97 |  | 0.002 |
| spo2 (mean (SD)) | 96.93 (1.89) | 97.08 (2.07) | 0.041 |  | 0.075 | 97.01 (1.87) | 97.03 (2.09) | 0.849 |  | 0.008 |
| atrial fibrillation (%) | 557 (36.9) | 661 (45.2) | <0.001 |  | 0.169 | 620.7 (40.8) | 603.5 (41.4) | 0.753 |  | 0.013 |
| chronic liver disease (%) | 68 (4.5) | 186 (12.7) | <0.001 |  | 0.296 | 141.0 (9.3) | 126.8 (8.7) | 0.697 |  | 0.019 |
| peripheral vascular disease (%) | 179 (11.9) | 229 (15.6) | 0.003 |  | 0.11 | 211.0 (13.9) | 196.0 (13.4) | 0.776 |  | 0.012 |
| myocardial infarct (%) | 229 (15.2) | 431 (29.4) | <0.001 |  | 0.348 | 346.8 (22.8) | 328.5 (22.5) | 0.898 |  | 0.006 |
| hypertension (%) | 839 (55.6) | 472 (32.2) | <0.001 |  | 0.483 | 661.0 (43.4) | 639.0 (43.9) | 0.83 |  | 0.009 |
| diabetes (%) | 484 (32.1) | 657 (44.9) | <0.001 |  | 0.266 | 577.4 (37.9) | 552.5 (37.9) | 0.999 |  | <0.001 |
| congestive heart failure (%) | 397 (26.3) | 619 (42.3) | <0.001 |  | 0.342 | 531.7 (34.9) | 509.8 (35.0) | 0.973 |  | 0.001 |
| dementia (%) | 114 (7.5) | 138 (9.4) | 0.077 |  | 0.067 | 130.7 (8.6) | 123.5 (8.5) | 0.926 |  | 0.004 |
| chronic pulmonary disease (%) | 308 (20.4) | 314 (21.4) | 0.51 |  | 0.026 | 315.9 (20.7) | 311.0 (21.3) | 0.724 |  | 0.015 |
| cancer (%) | 162 (10.7) | 203 (13.9) | 0.011 |  | 0.096 | 196.2 (12.9) | 187.4 (12.9) | 0.985 |  | 0.001 |
| charlson comorbidity index (median [IQR]) | 6.00 [4.00, 8.00] | 8.00 [6.00, 10.00] | <0.001 | nonnorm | 0.485 | 7.00 [5.00, 8.00] | 7.00 [5.00, 9.00] | 0.008 | nonnorm | 0.125 |
| sofa (median [IQR]) | 3.00 [1.00, 5.00] | 6.00 [4.00, 9.00] | <0.001 | nonnorm | 1.04 | 3.00 [2.00, 5.00] | 5.00 [3.00, 8.00] | <0.001 | nonnorm | 0.695 |
| lods (median [IQR]) | 3.00 [2.00, 5.00] | 6.00 [4.00, 8.00] | <0.001 | nonnorm | 0.819 | 4.00 [2.00, 6.00] | 5.00 [3.00, 8.00] | <0.001 | nonnorm | 0.524 |
| gcs (median [IQR]) | 15.00 [14.00, 15.00] | 15.00 [14.00, 15.00] | 0.002 | nonnorm | 0.044 | 15.00 [13.00, 15.00] | 15.00 [14.00, 15.00] | 0.004 | nonnorm | 0.067 |
| paraplegia (%) | 629 (41.7) | 416 (28.4) | <0.001 | nonnorm | 0.28 | 576.4 (37.9) | 469.4 (32.2) | 0.005 |  | 0.118 |
| renal disease (%) | 176 (11.7) | 623 (42.6) | <0.001 | nonnorm | 0.741 | 237.4 (15.6) | 517.0 (35.5) | <0.001 |  | 0.468 |
| wbc (median [IQR]) | 9.60 [7.30, 12.60] | 10.10 [7.40, 13.80] | 0.001 | nonnorm | 0.163 | 9.80 [7.32, 12.80] | 10.04 [7.40, 13.60] | 0.178 | nonnorm | 0.101 |
| rbc (median [IQR]) | 3.75 [3.18, 4.33] | 3.17 [2.75, 3.71] | <0.001 | nonnorm | 0.622 | 3.60 [3.10, 4.22] | 3.23 [2.79, 3.81] | <0.001 | nonnorm | 0.403 |
| hemoglobin (median [IQR]) | 10.80 [9.00, 12.70] | 9.30 [8.10, 11.00] | <0.001 | nonnorm | 0.54 | 10.40 [8.80, 12.20] | 9.59 [8.30, 11.32] | <0.001 | nonnorm | 0.276 |
| platelet (median [IQR]) | 250.00 [197.00, 321.00] | 178.00 [117.00, 245.00] | <0.001 | nonnorm | 0.715 | 254.00 [197.00, 334.00] | 178.00 [120.00, 240.00] | <0.001 | nonnorm | 0.75 |
| alt (median [IQR]) | 19.00 [13.00, 31.00] | 27.00 [15.00, 56.00] | <0.001 | nonnorm | 0.299 | 20.00 [13.00, 34.00] | 26.00 [15.00, 51.00] | <0.001 | nonnorm | 0.262 |
| ast (median [IQR]) | 23.00 [18.00, 35.00] | 37.00 [24.00, 70.00] | <0.001 | nonnorm | 0.27 | 25.00 [18.00, 37.00] | 35.41 [23.30, 62.00] | <0.001 | nonnorm | 0.24 |
| creatinine (median [IQR]) | 0.85 [0.70, 1.05] | 1.50 [1.05, 2.55] | <0.001 | nonnorm | 0.792 | 0.85 [0.70, 1.10] | 1.40 [1.00, 2.25] | <0.001 | nonnorm | 0.707 |
| inr (median [IQR]) | 1.20 [1.10, 1.40] | 1.30 [1.10, 1.50] | <0.001 | nonnorm | 0.255 | 1.20 [1.10, 1.40] | 1.20 [1.10, 1.50] | <0.001 | nonnorm | 0.094 |
| pt (median [IQR]) | 12.90 [11.80, 14.80] | 13.90 [12.50, 16.70] | <0.001 | nonnorm | 0.218 | 13.20 [12.00, 15.24] | 13.60 [12.30, 16.00] | 0.001 | nonnorm | 0.07 |
| ptt (median [IQR]) | 30.70 [27.20, 40.70] | 32.20 [27.80, 46.30] | <0.001 | nonnorm | 0.148 | 31.20 [27.50, 42.80] | 31.40 [27.50, 43.46] | 0.609 | nonnorm | 0.052 |
| chloride (median [IQR]) | 103.00 [100.00, 107.00] | 104.00 [99.00, 108.00] | 0.78 | nonnorm | 0.002 | 103.00 [100.00, 107.00] | 104.00 [100.00, 108.00] | 0.054 | nonnorm | 0.068 |
| potassium (median [IQR]) | 4.00 [3.70, 4.30] | 4.10 [3.80, 4.50] | <0.001 | nonnorm | 0.239 | 4.00 [3.70, 4.30] | 4.10 [3.80, 4.50] | <0.001 | nonnorm | 0.184 |
| calcium (median [IQR]) | 8.80 [8.30, 9.20] | 8.60 [8.10, 9.10] | <0.001 | nonnorm | 0.189 | 8.70 [8.30, 9.10] | 8.60 [8.10, 9.10] | <0.001 | nonnorm | 0.103 |
| sodium (median [IQR]) | 140.00 [137.00, 142.00] | 140.00 [136.00, 143.00] | 0.814 | nonnorm | 0.004 | 140.00 [137.00, 143.00] | 140.00 [137.00, 143.00] | 0.203 | nonnorm | 0.047 |
| los icu (median [IQR]) | 2.96 [1.56, 6.54] | 4.14 [1.94, 9.06] | <0.001 | nonnorm | 0.218 | 3.14 [1.55, 6.96] | 4.00 [1.87, 8.79] | <0.001 | nonnorm | 0.164 |
| los hospital (median [IQR]) | 9.86 [5.59, 18.08] | 13.62 [7.24, 24.19] | <0.001 | nonnorm | 0.273 | 11.19 [6.22, 19.91] | 12.79 [6.81, 23.30] | 0.001 | nonnorm | 0.143 |

**Note:** IPTW, Inverse Probability of Treatment Weighting; SMD, Standardized Mean Difference; SD, Standard Deviation; IQR, Interquartile Range; SOFA, Sequential Organ Failure Assessment; LODS, Logistic Organ Dysfunction System; GCS, Glasgow Coma Scale. Continuous variables are expressed as mean (SD) or median [IQR], depending on their distribution, while categorical variables are presented as frequencies and percentages (%). An absolute SMD < 0.1 indicates excellent balance of a covariate between the two groups. Of note, variables mathematically intrinsic to the EASIX calculation (i.e., platelets and creatinine), as well as comprehensive severity scoring systems incorporating these specific organ dysfunctions (e.g., SOFA and LODS), were purposely excluded from the propensity score model to prevent overadjustment and tautological bias. Consequently, as clinically expected, their SMDs appropriately remained > 0.1 following IPTW adjustment.

**Supplementary Table S4.** Predictive performance and optimal clinical cut-off values of lnEASIX for adverse mortality outcomes

| Outcomes | AUC | Optimal Cut-off | Sensitivity | Specificity |
| --- | --- | --- | --- | --- |
| 28-day Mortality | 0.66 | 0.67 | 0.58 | 0.66 |
| In-hospital Mortality | 0.67 | 1.01 | 0.51 | 0.75 |
| 90-day Mortality | 0.64 | 0.67 | 0.56 | 0.67 |
| 180-day Mortality | 0.64 | 0.63 | 0.56 | 0.66 |

**Note:** AUC, Area Under the Curve. The predictive performance of lnEASIX as a continuous variable was evaluated using Receiver Operating Characteristic (ROC) curves. The optimal clinical cut-off values for risk stratification across each temporal endpoint were determined by identifying the maximum Youden Index (Sensitivity + Specificity - 1).

**Supplementary Figure S1.** Covariate balance check before and after Inverse Probability of Treatment Weighting (IPTW)

Absolute standardized mean differences (SMDs) of baseline covariates between the low and high lnEASIX groups before (red circles, unadjusted) and after (blue triangles, adjusted) the application of IPTW. Panels (A-D) illustrate the balance diagnostics for the specific cohorts analyzed across the 28-day, in-hospital, 90-day, and 180-day mortality endpoints, respectively. The dashed vertical line denotes the stringent threshold for optimal covariate balance (SMD = 0.1). Following IPTW adjustment, all included variables demonstrated an SMD < 0.1, indicating successful mitigation of baseline confounding

**Supplementary Figure S2.** IPTW-adjusted Kaplan-Meier cumulative survival curves stratified by median lnEASIX.

IPTW-adjusted cumulative survival probabilities from the day of admission for (A) 28-day, (B) 90-day, and (C) 180-day observation periods. Patients were dichotomized into low (blue line) and high (red line) lnEASIX groups based on the overall median value. The high lnEASIX group consistently exhibited significantly worse survival across all temporal endpoints. Statistical differences between the groups were evaluated using weighted Cox proportional hazards regression models with robust variance estimators (all Weighted P < 0.001).

**Supplementary Figure S3.** **Nomogram for predicting the probability of 28-day, 90-day, and 180-day mortality in critically ill patients with ischemic stroke.**

The nomogram was developed by incorporating lnEASIX, age, Sequential Organ Failure Assessment (SOFA) score, and CCL. To use the nomogram, locate the patient's value for each variable on its respective axis, and draw a vertical line upwards to the "Points" axis to determine the score for that variable. Sum the points for all four variables to obtain the "Total Points". Finally, draw a vertical line downwards from the "Total Points" axis to estimate the individual's probability of mortality at 28, 90, and 180 days.

**Supplementary Figure S4. Decision curve analysis (DCA) evaluating the clinical net benefit of integrating lnEASIX with established scoring systems**

**A-D** DCA curves demonstrate the clinical utility of adding lnEASIX to baseline SOFA, LODS, and GCS models for predicting **A** in-hospital, **B** 28-day, **C** 90-day, and **D** 180-day mortality. The x-axis represents the continuous threshold probability for predicting mortality, and the y-axis represents the calculated net clinical benefit. The solid green horizontal line assumes that no patients experience the event and no intervention is performed (Treat None, net benefit = 0). The solid red line assumes that all patients experience the event and receive intervention (Treat All). The blue lines denote the net benefit of relying solely on the baseline clinical scores (SOFA, LODS, or GCS alone). The purple lines represent the net benefit of the integrated models incorporating lnEASIX (e.g., SOFA + lnEASIX). Across all evaluated temporal endpoints, the integrated models (purple lines) consistently yielded a higher net benefit than the baseline clinical scores alone (blue lines) across a broad spectrum of clinically relevant threshold probabilities, confirming the real-world utility of lnEASIX for optimizing prognostic risk stratification.
